# Repurposing of Plasminogen: An Orphan Medicinal Product Suitable for SARS-CoV-2 Inhalable Therapeutics

**DOI:** 10.3390/ph13120425

**Published:** 2020-11-27

**Authors:** Anna Maria Piras, Ylenia Zambito, Maurizio Lugli, Baldassare Ferro, Paolo Roncucci, Filippo Mori, Alfonso Salvatore, Ester Ascione, Marta Bellini, Roberto Crea

**Affiliations:** 1 Department of Pharmacy, University of Pisa, Via Bonanno 33, 56126 Pisa, Italy; ylenia.zambito@unipi.it; 2 Alfatest Srl, Via Pellizza da Volpedo 59, 20092 Cinisello Balsamo, Milano, Italy; maurizio.lugli@alfatest.it; 3 Anestesia e Rianimazione, Azienda USL Toscana Nord Ovest, Viale Alfieri 36, 57124 Livorno, Italy; baldoferro81@gmail.com (B.F.); paolo.roncucci@uslnordovest.toscana.it (P.R.); 4 Kedrion S.p.A., Via di Fondovalle, Loc. Bolognana, 55027 Gallicano, Italy; F.Mori@kedrion.com (F.M.); A.Salvatore@kedrion.com (A.S.); e.ascione@kedrion.com (E.A.); M.Bellini@kedrion.com (M.B.); R.Crea@kedrion.com (R.C.)

**Keywords:** SARS-CoV-2, COVID-19, human plasminogen, inhalation, aerosol, coagulopathy, ARDS, orphan drug

## Abstract

The SARS-CoV-2 infection is associated with pulmonary coagulopathy, which determines the deposition of fibrin in the air spaces and lung parenchyma. The resulting lung lesions compromise patient pulmonary function and increase mortality, or end in permanent lung damage for those who have recovered from the COVID-19 disease. Therefore, local pulmonary fibrinolysis can be efficacious in degrading pre-existing fibrin clots and reducing the conversion of lung lesions into lasting scars. Plasminogen is considered a key player in fibrinolysis processes, and in view of a bench-to-bedside translation, we focused on the aerosolization of an orphan medicinal product (OMP) for ligneous conjunctivitis: human plasminogen (PLG-OMP) eye drops. As such, the sterile and preservative-free solution guarantees the pharmaceutical quality of GMP production and meets the Ph. Eur. requirements of liquid preparations for nebulization. PLG-OMP aerosolization was evaluated both from technological and stability viewpoints, after being submitted to either jet or ultrasonic nebulization. Jet nebulization resulted in a more efficient delivery of an aerosol suitable for pulmonary deposition. The biochemical investigation highlighted substantial protein integrity maintenance with the percentage of native plasminogen band > 90%, in accordance with the quality specifications of PLG-OMP. In a coherent way, the specific activity of plasminogen is maintained within the range 4.8–5.6 IU/mg (PLG-OMP pre-nebulization: 5.0 IU/mg). This is the first study that focuses on the technological and biochemical aspects of aerosolized plasminogen, which could affect both treatment efficacy and clinical dosage delivery. Increasing evidence for the need of local fibrinolytic therapy could merge with the availability of PLG-OMP as an easy handling solution, readily aerosolizable for a fast translation into an extended clinical efficacy assessment in COVID-19 patients.

## 1. Introduction

The SARS-CoV-2 infection (also known as COVID-19) is associated with the development of acute respiratory distress syndrome (ARDS). ARDS is present in approximately 75% of patients in need of intensive care and is recognized as the main cause of mortality [1]. The pathophysiology of ARDS includes an acute inflammation at alveolar level, followed by coagulopathy (coagulation and fibrinolytic systems dysregulation) with local deposition of fibrin, both in the alveolar spaces and in the lung parenchyma. Thus, a hyaline membrane is formed, consisting of fibrin meshes entrapping plasma proteins and cellular fragments, which prevents gas exchange during respiration [2].

The scientific community firmly believes that thrombolytic action against fibrin depots in the lungs could be a powerful key to improving the ventilation–perfusion ratio of COVID-19 patients [3]. Indeed, while the administration of anticoagulant drugs prevents the formation of new clots, there is no effect on pre-existing lung fibrin deposits. Instead, this latter effect is attributable to fibrinolytic drugs, such as plasminogen activators. A Tissue Plasminogen Activator (t-PA) is currently under clinical investigation for COVID-19 treatments [4,5,6,7], but being mainly subject to parenteral administration, it carries a high risk of systemic bleeding [8]. For these reasons, the inhalation route could limit systemic exposure to fibrinolysis, providing a pulmonary loco-regional action and representing a new therapeutic opportunity for patients suffering from COVID-19 [2].

Plasminogen is a key component of the endogenous fibrinolytic system. It is a single chain plasma protein (MW 92 kDa), and it is the inactive precursor (zymogen) of the serine protease plasmin (fibrinolytic enzyme). The conversion of plasminogen into plasmin occurs in nature under the action of the plasminogen activators, t-PA and u-PA. The formed plasmin cleaves the clots into soluble degradation products. Furthermore, plasminogen acts as a regulator in physiological processes such as wound healing [9,10] and as an immunomodulator in inflammatory processes [11,12,13].

Therefore, the presence of plasminogen at pulmonary level could play a dual active role against the pathophysiology of ARDS, counteracting both the acute alveolar inflammation and lysing the clots resulting from pulmonary microcoagulopathy. Additionally, although patients at an early stage of COVID-19 infection present normal levels of plasminogen, patients progressing towards severe ARDS symptoms demonstrate lower plasminogen values due to its consumption of fibrinolysis activation [14].

In the epidemiological emergency context generated by the SARS-CoV-2 infection, the administration of plasminogen in COVID-19 patients was subject to preliminary clinical evaluation, coordinated by the Talengen Institute of Life Sciences [15]. The study highlighted the positive effects obtained from inhalation of plasminogen, with significant improvements to pulmonary lesions and increased oxygen saturation in patients under moderate-critical conditions.

Indeed, the administration by inhalation route permits direct delivery of the drug to the site of action, thus favoring the rapid onset of therapeutic effects. Pulmonary administration allows for the achievement of high local drug dosages, limiting systemic toxicity and side effects, which may occur with parenteral administration [16]. Similarly, the loco-regional administration of plasminogen could provide targeted, non-generalized action, which would not be achievable by the parenteral route.

Administration by inhalation is strictly related to the use of medical devices, such as nebulizers, pressured inhaler, etc. Among these, the simplest way to introduce a drug into clinical practice is to aerosolize it starting from its aqueous solution by using continuous nebulization. Presently, there are three main nebulization technologies, i.e., pneumatic (jet nebulization), ultrasonic, and using a microperforated vibrating membrane. Each device is designed to produce aerosols with controlled droplet size distributions to reach different segments of the respiratory tract. However, every nebulization technique causes shear stress and heat, which can affect drug physiochemical properties, thus precluding its therapeutic action [17]. It is thus mandatory to evaluate the nebulization of the drug dosage form in order to establish its suitability to nebulization, both in terms of aerosol droplet size distribution and drug stability upon nebulization. Among the three technologies, the vibrating mesh is the most recent and more expensive, less aggressive towards labile drugs, typically with a medium size mesh of 5 μm, and still not widespread. Jet nebulizers are considered the gold standard for delivering drugs to hospitalized patients in clinical settings, yet mechanical shear forces can damage the delivered drugs. As for ultrasonic nebulizers, they are often associated with high drug degradation due to both the shear stress at the air-liquid interface and the cavitation mechanism, as well as the dissipation of energy in the form of heat. This latter technique is considered deleterious for protein drugs, which are subject to thermal unfolding and denaturation [17,18].

Since different nebulization technologies could provide diverse harmful stresses and aerosol outputs, it is necessary to collect preclinical technological information on the appropriate choice of nebulizer as a function of drug product and therapy [19].

In the present paper, jet and ultrasonic nebulization were evaluated for the pulmonary delivery of plasminogen by using a pharmaceutical grade homogeneous aqueous solution of the plasma derived protein. Presently, pharmaceutical grade plasma-derived plasminogen (PLG) is available as eye drops (Kedrion S.p.A.) and currently used as an orphan medicinal product (OMP), according to Italian law 648/96, for the treatment of ligneous conjunctivitis. The conceptualization of the research considers that the requirements of the European Pharmacopoeia (Ph. Eur.) 10th edition for inhaled solutions are similar to those for eye drops (solutions) and that the dosage form prepared according to GMP standards guarantees the safety of pharmaceutical quality for a ready translation to clinic.

## 2. Results

### 2.1. Physical Characterization

The physicochemical properties of PLG-OMP and isotonic PLG-OMP solutions (iPLG-OMP) were evaluated and are shown in Table 1. Solution density, viscosity and surface tension were determined and the values compared to those of water and physiologic saline solution (NaCl 0.9%). The latter was also used as a reference for the nebulization assays. PLG-OMP solutions have physical properties similar to those of NaCl 0.9%, including surface tension value. As expected, iPLG-OMP has physical properties similar to those of PLG-OMP, differing only in the osmolality value, which was intentionally corrected to 300 mOsm/kg H_2_O.

Solution properties are known to affect both jet and ultrasonic nebulization [20]. Concerning the viscosity, low values such as those of PLG-OMP and iPLG-OMP are generally preferred in order to gain higher aerosol outputs and generate smaller droplets. Solution density mainly affects the aerodynamics of droplets and, as consequence, their impact and deposition in the respiratory tract, with increasing density leading to droplets impacting the upper airways [21]. The density of the droplets may vary during transportation through the airways, mainly because of superficial phenomena, namely surface solvent evaporation or condensation of airway water vapor. These are correlated to hydrophobicity of the dissolved compound and liquid osmolality. Concerning PLG-OMP and iPLG-OMP, the presence of NaCl mainly governs these effects. The concentration of NaCl affects the extent of relative humidity (RH) near the surface of the droplets, and high NaCl concentrations result in decreased water vaporization and increased water condensation. These effects are generally balanced in isotonic conditions, although they can still be affected by airways airflow and therefore by the presence of pathological conditions [22]. For these reasons, the similarity of PLG-OMP and iPLG-OMP with 0.9% NaCl saline is fundamental in view of future aerosolization in a clinical setting. It should also be added that saline NaCl 0.9% is commonly applied for in vitro/in vivo and in vitro/ex vivo correlations, aimed at determining the distribution of droplets in the airways. Those are typically carried out by the nebulization of a physiological solution containing small quantities of radioactive tracer (i.e., ^99m^Tc) [23]. Furthermore, isotonic saline solutions, and sometimes even hypertonic ones, are commonly administered to relieve breathlessness in patients affected by chronic obstructive pulmonary disease (COPD), infectious pneumonia and cystic fibrosis [24]. Therefore, the similarity of PLG-OMP and iPLG-OMP solutions with NaCl 0.9% suggests their suitability for nebulization.

### 2.2. Nebulization and Biochemical Characterization

Both PLG-OMP and iPLG-OMP samples underwent nebulization procedures. Two commercial devices with different nebulization technologies were applied, namely the jet nebulizer OMRON A3 complete (flow setting within 2–15 L/min) and the ultrasonic device, PIC airproject plus. Despite having different operating principles, the two devices are meant to provide similar Mass Median Aerodynamic Diameter (MMAD) aerosols, as declared by manufacturers, particularly referring to the ampule position 3 of the jet nebulizer (called J3) and the PIC ultrasonic nebulizer (US). Concerning the ampule position 2 of the jet nebulizer (called J2), it generates aerosols with a higher MMAD and is more suitable for the treatment of tracheo-bronchial pathologies, as declared by manufacturer and in line with general correlation between MMAD and the deposition in the respiratory tract [25]. Despite being widely spread, jet and ultrasonic devices can both affect protein stability upon nebulization due to high flow share stress or heat generation, respectively [20]. The effect of US, J3 and J2 settings on plasminogen stability was performed by biochemical characterization of aerosolized PLG-OMP and iPLG-OMP samples. As for nebulization rates, the three nebulization settings recorded J2 0.47 ± 0.01 mL/min, J3 0.89 ± 0.09 mL/min and US 0.56 ± 0.13 mL/min, respectively, in line with what was expected from the device specifications.

Aliquots of aerosolized PLG-OMP and iPLG-OMP were collected and submitted for biochemical characterization. The same analyses were performed on not-aerosolized control solutions (plain PLG-OMP and iPLG-OMP samples) as well as on the residual solutions collected from the device ampoules at the end of the nebulization. The stability of the aerosolized protein toward the nebulization processes, starting from either PLG-OMP or iPLG-OMP samples, was related to several parameters, including autoactivation of plasminogen to plasmin, the formation of Lys-plasminogen (I and II), the presence of protein fragments or aggregates and the maintenance of plasminogen-specific activity.

Plasminogen is the zymogen of the plasmin enzyme. Under stressful conditions, the zymogen can activate itself and exert a catalytic function by activating other plasminogen molecules. Therefore, the presence of an activated enzyme is generally considered an important stability issue [26,27]. In our case, it can be a signal of the instability of the protein formulation when it is submitted for the aerosolization process. As shown in Table 2, the stress suffered by the samples was not such as to activate the plasminogen as no plasmin activity was detected, regardless of the applied nebulization technology.

SDS-PAGE of aerosolized PLG-OMP and iPLG-OMP samples was performed in order to provide further evidence of the structural integrity of the protein in the nebulized samples, as well as detect the eventual formation of fragments. Data from three batches of nebulized products proved that over 90% were structurally intact plasminogen (in agreement with the specification of the finished PLG-OMP product), as shown in Table 2. The remaining percentage was due to the presence of process impurities. LC-MS/MS analysis (data not shown) demonstrated that a small number of impurities (about 2–3%), mainly Immunoglobulins and Albumin, were associated with the product and derived mainly from the plasma used for purification. An exception was observed for iPLG-OMP, which was aerosolized by using an ultrasonic device. In this latter case, % PLG-band was reduced below 90%, indicating a more harmful effect on the protein.

**Figure 1:**
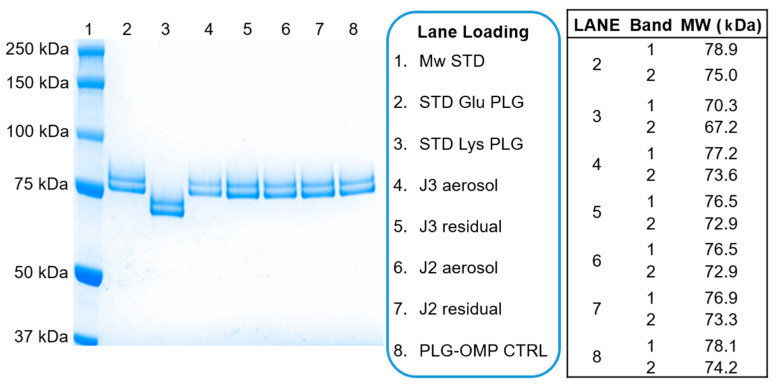
Lys-PLG SDS-PAGE represents 8% of the Bis-Tris gel of control and jet nebulized PLG-OMP samples. Stained gel, lane loading and bands Mw are reported from left to right, respectively.

Glu-PLG is the native form of plasminogen. The prefix Glu is due to the presence of glutamic acid as the first amino acid of the chain. Lys-PLG is the most common truncated form of plasminogen, missing the first 77 amino acids and displaying lysine as the first amino acid of the chain. The properties of Lys-PLG are different from Glu-PLG, especially in the readiness of activation. Lys-PLG within the PLG-OMP product and iPLG-OMP solutions is considered an impurity or an unwanted pre-activation state; the presence of Lys-PLG in aerosolized samples would reveal the occurrence of aggressive stress. Under specific electrophoretic conditions, the Glu-PLG migrates as two separate bands corresponding to the two glycoforms: Glu I and Glu II. If present, the Lys-PLG is visualized as two separate bands deriving respectively from Glu I and Glu II and which are named Lys I and Lys II (Figure 1).

In all the analyzed samples, the percentage of protein bands referable to Lys-Plasminogen were found to be 0%, regardless of the applied nebulization procedure. In all samples, it was possible to appreciate the protein bands attributable to Glu-PLG forms as indirect confirmation of the previously described results (Figure 1, Table 2).

Plasminogen-specific activity was measured in all aerosolized samples and compared with control PLG-OMP and iPLG-OMP solutions.

**Figure 2:**
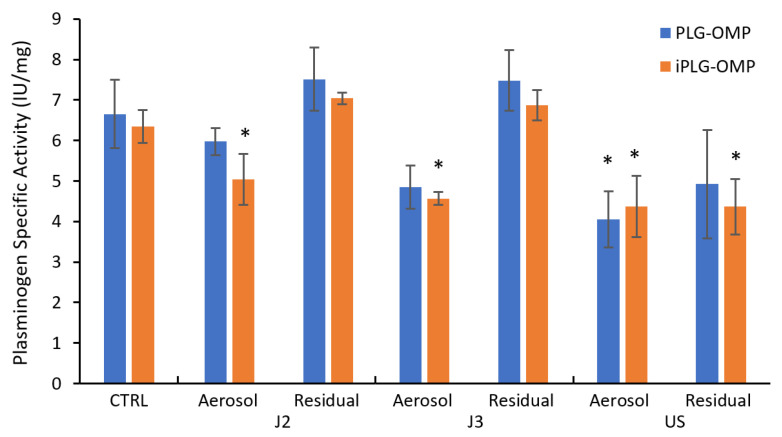
Jet (J2 and J3) and ultrasonic (US) nebulization of PLG-OMP and iPLG-OMP solutions. Specific activity of plasminogen was determined in the aerosol and residual solutions, which were collected from the ampoules at the end of nebulisation. The data were statistically analyzed using Student’s t-test. Statistical significance was set at the level of * *p* < 0.01.

Both PLG-OMP and iPLG-OMP solutions were analyzed using the Plasminogen potency evaluation, and the protein concentration was measured by the Bradford protein assay. Subsequently, the specific activity was calculated and compared with those of the sample Control (Figure 2).

Enzymatic activity was found in the three batches of PLG Samples analyzed after nebulization, reflecting the fact that the protein, in addition to structural integrity, also maintained total/partial functional integrity. The nebulization of both PLG-OMP and iPLG-OMP solutions involves a modest loss of specific activity, compared with the control solutions. In particular, the specific activity of aerosol samples is higher for pneumatic nebulization and lower for ultrasonic nebulization, according to the J2 > J3 > US trend. The PLG-OMP samples nebulized with Omron Jet, in particular the ones using position 2, provided specific activity very similar to that of the Control samples. This evidence of the functional integrity of the product after nebulization would make this condition preferable.

Despite maintaining specific activity in the range 4.5–5.0 IU/mg, the dilution carried out to prepare iPLG-OMP increased the susceptibility of plasminogen to nebulization.

It should be noted that the part of the product that was not nebulized was identified as a residue. PLG-OMP and iPLG-OMP Residual samples maintained functional integrity. In fact, the specific activity of these samples was comparable to that of the controls, with the exception of the residue of the ultrasonic samples, where the specific activity showed a significant reduction, confirming a greater aggressiveness of the ultrasonic setting on protein stability. These data are in agreement with those observed using the SDS-PAGE method.

### 2.3. Droplet Size Distribution

**Figure 3:**
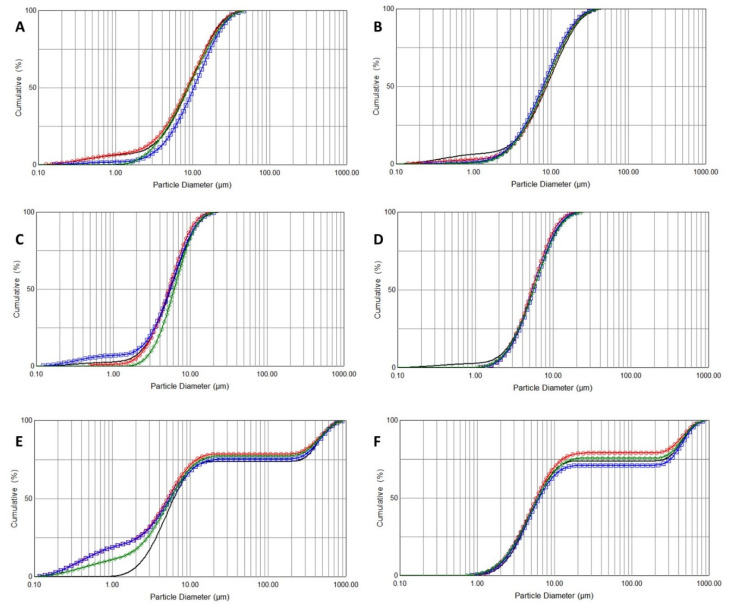
Cumulative particle size distribution (PSD) of aerosolized products by laser diffraction evaluation, the overlay of three sample repetitions (red, blue and green lines) and NaCl 0.9% saline solution, used as reference (black line). (**A**,**B**) J2 jet nebulization of PLG-OMP and iPLG-OMP, respectively; (**C**,**D**) J3 jet nebulization of PLG-OMP and iPLG-OMP, respectively; (**E**,**F**) US ultrasonic nebulization of PLG-OMP and iPLG-OMP, respectively.

To determine the droplet size distribution of the aerosols, laser diffraction measurements were performed. As displayed in Figure 3, the aerosols obtained for both PLG-OMP and iPLG-OMP have particle size distribution (PSD) superimposable on that obtained for the NaCl 0.9% reference solution, mostly irrespective of the applied nebulization technique. Considering that the physical properties of PLG-OMP and iPLG-OMP are similar to those of the physiological saline solution, the results obtained by laser diffraction confirm what was expected. No significant difference was observed between PLG-OMP and iPLG-OMP when submitted to the same nebulization setting (Table 3).

However, it should be noted that the aerosol produced by US nebulization is not homogeneous over time, as observed by monitoring the Transmission value of detector 0 during the entire nebulisation period (data not reported). The results evidenced a heterogeneous polymodal size distribution, displaying droplets with median diameter values differing by two orders of magnitude. This behaviour is recorded for both the reference physiological solution and the samples, this being the limit of device performance. The wide polydispersion obtained with US nebulization is confirmed by the high SPAN value (Table 3).

Concerning the jet nebulizations, the generated aerosols have a narrow particle size distribution, with small SPAN values and median diameters analogous to those obtained for the NaCl reference solution. Additionally, these parameters fall within the range indicated by the device manufacturer. It is also worth noting that the percentages of droplets below 10 μm and 5 μm are strictly dependent on nebulization settings and not on the nebulised solution sample, since both PLG-OMP and iPLG-OMP share similar physical properties with the NaCl reference solution. In particular, lower air flow position (J2) corresponds with bigger dv_(50)_, with about 60% and 25% of droplets sized below 10 μm and 5 μm, respectively. Better yield is obtained by the J3 setting, which provides higher shear stress and, consequently, lower-sized particles, resulting in more than 80% and more than 40% of droplets being sized below 10 μm and 5 μm, respectively.

## 3. Discussion

From a physical point of view, both types of samples (original lots and isotonic samples) comply with the general specifications reported in FUXII for liquid preparations for nebulization. Both are homogeneous solutions, their pH (pH 6.5) falls within the required pH range 3.0–8.5 and, being sterile, they satisfy microbiological requirements. Furthermore, the data obtained do not significantly deviate from the 0.9% saline solution values, suggesting a good nebulization yield in agreement with the specifications of the nebulization devices. According to what is reported in the literature, the physical characteristics detected are in line with the data of the formulations used for treatment of the lower airways [28]. The droplet size distribution obtained by applying continuous nebulization processes to aqueous solutions depends mainly on device performance. Indeed, since PLG-OMP, iPLG-OMP and NaCl 0.9% have similar physical features, the obtained aerosols are homogeneous, and the differences in size distribution can be minimized.

Furthermore, since the nebulized solutions are homogenous and have physical properties analogous to those of NaCl 0.9%, the size distribution obtained by laser diffraction analysis could be assimilated into the aerodynamic distribution of the droplets [29,30]. This means that with the pneumatic device J2 settings, the MMDA of the droplets is compatible with the treatment of tracheo-bronchial pathologies, with only about 25% of the aerosol capable of reaching the lower airways tract [31]. Conversely, with the pneumatic device J3 settings, the MMDA of the aerosol is suited to the treatment of bronchopulmonary diseases, with more than 40% of droplets being smaller than 5 μm [31].

To support the inherent feasibility of the nebulization of Plasminogen, it is important to underline that meticulous biochemical analysis highlighted the functional and structural integrity of the product. Moreover, no indicator of product degradation has been observed.

In particular, the Protein composition (SDS-PAGE) did not show the presence of low molecular weight fragments or aggregates in any of the analyzed samples. The presence of Lys Plasminogen was also not found in any of the analyzed samples, reflecting the fact that the sample did not undergo any pre-activation. All tests carried out seem to be in tune and confirm each other’s results. However, a part of the product, called Residue because it was not nebulized, remained active and showed no signs of degradation. More sensitive chromatographic methods (size exclusion chromatography, ion exchange chromatography) as well as dynamic light scattering analysis (DLS) will be set for future corroboration of the presently acquired data. Such methodologies are frequently applied in parallel with biochemical investigation in order to assess protein stability during the optimization of inhalable formulations [32].

Jet nebulization was less aggressive towards the nebulized plasminogen, particularly for the J2 mode, which is characterized by a more rapid nebulization. However, the J3 setting also allowed for good maintenance of plasminogen stability and, considering the higher percentage of aerosol respirable fraction, it is possible to conclude that this setting is adequate for the treatment of bronchopulmonary pathologies.

Indeed, the collected data represent a starting point for deeper investigations, including the ability of aerosolized plasminogen to reach the low respiratory tract and eventually pass the alveolar capillary membrane [33]. The goal is to transfer the drug to the site of action, concentrating the local effect and minimizing the collateral effect due to systemic infusion. Such effects have already been described in the literature, even for more labile and larger plasma derived proteins such as immunoglobulins [28]. Additionally, even though it was collected by administering a different formulation, the local effect of inhaled PLG has been reported [15], highlighting the capability of plasminogen to exert fibrinolytic local action. The data collected until now did not allow for the precise individuation of a clinical dosage for inhalable PLG-OMP but rather assessed the activity maintenance of the orphan drug upon nebulization, which appeared promising for its translation into COVID-19 clinical treatment. In fact, the control of hypercoagulation and the fibrinolytic pathway seems to be a promising way to improve oxygenation and gas diffusion in COVID-19 pneumonia and other pathologies in which ventilation/perfusion mismatch and right to left shunt are predominant [2]. Additionally, the setting of short-term clinical treatment would reduce the probability of harmful immune response [34], which is a relevant aspect to consider for protein-based therapeutics [35].

A similar approach is presently under investigation in COVID-19 patients with nebulized heparin as an anticoagulant agent [36], which is also known to reduce inflammation by modulating alveolar macrophages [37,38].

## 4. Materials and Methods

### 4.1. Materials

Human plasminogen eye drops 1 mg/mL (PLG-OMP, Orphan Medicinal Product number EU/3/07/461 in EU and Orphan Designation number 10-3092 in US), Kedrion S.p.A.; excipients water and NaCl); saline solution NaCl 3% (Libernar^®^, Laboratorie Unither, Amiens, France), saline solution NaCl 0.9% (Eurospital, Trieste, Italy), water for HPLC (Sigma-Aldrich, St. Louis, MO, USA). The Plasminogen Kit (IL) consists of lyophilized chromogenic substrate S-2403, pyroGlu-Phe-Lys-pNA.HCl (4.2 mg/vial) and bulking agent; the streptokinase reagent (20,000 U/vial), fibrinogen, and human serum albumin. HemosIL Calibration Plasma (Werfen, Barcelona, Spain) and HemosIL Normal Control ASSAYED (Werfen), HemosIL Factor Diluent (Werfen); 4th international standard for Plasmin (NIBSC), MiniGel (NuPAGE); LDS Sample Buffer 4× (NuPAGE), Antioxidant (NuPAGE); MOPS SDS Running Buffer 20× (NuPAGE); Bis-Tris Precast Gels (NuPAGE); Simply Blue Safe Stain, Molecular Weight Standards (BioRad “All Blue”, Hercules, CA, USA); Glu-Plasminogen Standard (Kedrion, Barga, Italy); Lys-Plasminogen Standard (Sigma-Aldrich); Albumin Standard (Kedrion); water for HPLC (Sigma-Aldrich); Coomassie Protein Assay Reagent (PIERCE, Waltham, MA, USA).

### 4.2. Methods

#### 4.2.1. Preparation of Isotonic PLG (iPLG)

Isotonic PLG-OMP solutions (iPLG-OMP) were prepared by the simple dilution of 6 parts of PLG-OMP with 1 part of Libernar^®^ solution (i.e., 2 mL of NaCl 3% were added to 12 mL of PLG-OMP).

#### 4.2.2. Nebulization

Aliquots of 5 mL (PLG-OMP and iPLG-OMP specimens) were atomized using either ultrasonic ultrasonic airproject plus (PIC) or pneumatic (Jet) OMRON A3 complete (OMRON). In the case of pneumatic nebulization, two delivery modes, position 2 and 3 of the bulb, were adopted. Nebulization was conducted until no cloud was produced. Atomized samples were collected by condensation into a glass bioaerosol impinger (0–4 °C) and stored at −20 °C until analysis [39]. Both the residual solutions in the medication reservoir and the collected nebulized aliquots were submitted to biochemical characterization and compared with the untreated PLG solutions. Untreated PLG solutions, named CTRL samples, were submitted to freeze thawing, similar to aerosolized specimens.

#### 4.2.3. Physical Characterization

Both PLG-OMP and iPLG-OMP underwent extensive physical characterization, as described below. pH measurements were performed using pHmetro Orma electronic p200; solution density was evaluated using the hydrostatic method (Mettler AE163, Columbus, OH, USA) calibrated with water for HPLC use; osmolality was measured by a Roebling osmometer (calibrated with NaCl 300 mOsm/kg H_2_O), cinematic viscosity was determined by a Cannon-Fenske (50) viscosimeter under ASTM D-445 standard procedure, using water for HPLC use as a reference(Cannon, State College, PA, USA); liquid surface tension was measured using a Force Tensiometer (KRŰSS-K6, KRŰSS, Hamburg, Germany). All measures were performed under controlled temperature (22 ± 1 °C).

#### 4.2.4. Biochemical Analytical Methods

All biochemical characterizations were performed under standardized procedures, in accordance with ICH Topic Q2 (R1) [40].

- Protein quantification

Protein concentration was determined spectrophotometrically according to the Bradford methods [41] with Biorad protein reagent. A plasminogen pool was used as the standard.

- Plasminogen specific activity

Plasminogen activity (PLG:Act) was quantified by a Hemosil Plasminogen test-kit (Instrumentation-Laboratory) according to manufacturer’s instructions. A Hemosil calibration plasma kit was used for calibration. Plasminogen specific activity (IU/mg) was calculated referring to the protein concentration determined for control and aerosolized specimens.

- Plasmin activity (PL:Act)

Stress-induced conversion of plasminogen to plasmin was evaluated by measuring plasmin activity (PL:Act) in aerosolised samples. PL:Act was quantified by using a lyophilized chromogenic substrate (S-2403, pyroGlu-Phe-Lys-pNA HCL 8 mg/vial) from a Hemosil Plasmin Inhibitor test-kit (Instrumentation-Laboratory) according to the manufacturer’s instructions. International Standard Plasmin Concentrate (13/206, NIBSC) was used as calibrator. The p-nitroaniline released from the chromogenic substrate was kinetically monitored at 405 nm and was directly proportional to the quantity present in the analyzed sample.

- Protein characterization

Native glutamic acid-plasminogen (Glu-PLG) and Lysine-plasminogen (Lys-PLG) were determined by SDS-PAGE. *Glu-PLG.* SDS-PAGE was carried out under denaturing conditions using 4–12% gel (Invitrogen, Carlsbad, CA, USA). Plasminogen and home-made PLG standard samples were prepared using an LDS sample buffer 4× (invitrogen) as a denaturing agent, followed by thermal denaturation at 95 °C for 5 min. 5 µg of protein PLG samples were loaded onto the gel, and the electrophoretic run lasted 55 min at 200 volts. In a second test, the gel was stained with Simply Blue Safe protein stain (Invitrogen) for 1 h and subsequently destained with water. The gel was acquired at Chemidoc MP using ImageLab software Lys-PLG. SDS-PAGE was carried out under denaturing conditions using 8% gel (Invitrogen). Plasminogen and Lys-PLG standard samples were prepared using an LDS sample buffer 4× (Invitrogen) as a denaturing agent, followed by thermal denaturation at 95 °C for 5 min. 1 µg of protein PLG samples were loaded onto the gel, and the electrophoretic run lasted 55 min at 200 volts. In a second test, the gel was stained, destained and acquired as previously described.

The results were expressed as % of the band attributed to Glu-PLG or Lys-PLG, with respect to all bands present in the Line (impurities, degradation products, etc.).

#### 4.2.5. Droplet Size Distribution

Droplet size distribution measurements were conducted at Alfatestlab S.r.l (Cisanello Balsamo, Milan, Italy) using Malvern Panalytical’s Spraytec laser diffraction system (Malvern, Instruments, Worcestershire, UK). 5 mL of each PLG-OMP and iPLG-OMP solution was nebulized by applying the mouth mask of each nebulization apparatus, located at 80 ± 2 mm from the laser cell. Measurements were performed according to ISO 13320:2009 standards at 23 ± 2 °C and a relative humidity of 50 ± 5%.

#### 4.2.6. Statistical

PLG-OMP specimens were collected from three product batches, and the same batches were used for the preparation of iPLG-OMP samples. Each analytical test was conducted at least in triplicate on each batch sample. When possible, the data sets were statistically compared by applying a Student *t*-test.

## 5. Conclusions

The study assesses the feasibility of delivering inhalable plasminogen starting from human plasminogen orphan medicine (PLG-OMP). The research has shown that, even if none of the tested conditions massively degraded plasminogen, the ultrasonic and jet nebulization of PLG-OMP were not interchangeable. It is also concluded that PLG-OMP can be nebulized as it is. Additionally, if it would be necessary to dilute PLG-OMP to iPLG-OMP within a clinical setting, it can still be nebulized, although a lower activity of the drug should be expected.

Considering the recent approval of clinical trials based on the inhalation of fibrinolytic drugs such as t-PA [42], the present study opens up the opportunity for adjunctive therapy regimes based on the repurposing of PLG-OMP as inhalable medicine for the treatment of ARDS in COVID-19 patients. The acquired preclinical data appear useful for the bench-to-bedside translation of PLG-OMP in the treatment of COVID-19 patients, in view of evaluating safety issues and fine dosage/efficacy correlation.

## Figures and Tables

**Table 1:** Physical properties of human plasminogen (PLG-OMP) and iPLG-OMP solutions evaluated at (22 ± 2 °C).

Physical Parameter	PLG-OMP	iPLG-OMP	H_2_O	NaCl 0.9%
pH	6.6 ± 0.3	6.4 ± 0.2	6.9 ± 0.1	6.0 ± 0.2
density g/mL	0.9710 ± 0.0060	0.9834 ± 0.0048	0.9972 ± 0.0234	1.0051 ± 0.0226
Osmolality mOsm/Kg H_2_O	205 ± 10	316 ± 12	-	300 ± 4
Kinematic Viscosity cSt	0.969 ± 0.010	0.981 ± 0.008	0.957 ± 0.008	1.010 ± 0.008
Dynamic Viscosity cP	0.941 ± 0.007	0.965 ± 0.007	0.954 ±0.002	1.015 ± 0.024
Surface Tension * mN/m	62.7 ± 0.6	63.0 ± 0.2	71.5 ± 0.6	62.8 ± 0.8

* Measurements were performed at 25 ± 2 °C; hypertonic saline solution NaCl 3.0% (Libernar^®^) 72.0 ± 0.2 mN/m.

**Table 2:** Biochemical characterization of PLG-OMP and iPLG-OMP solutions before (CTRL) and after aerosolization under the three nebulization settings: jet nebulizations (J2 and J3) and ultrasonic nebulization (US); both aerosol and ampoule residual solutions are displayed. All values are reported as the mean of at least three repetitions (mean ± SD).

		CTRL	J2		J3		US	
			Aerosol	Residual	Aerosol	Residual	Aerosol	Residual
PLG-OMP	PL:Act (U/mL)	<0.005	<0.005	<0.005	<0.005	<0.005	<0.005	<0.005
	%PLG-band	95.6 ± 2.2	92.2 ± 1.6	95.0 ± 2.3	91.4 ± 0.4	94.0 ± 0.3	90.8 ± 2.2	92.9 ± 2.9
	% GLU I	45.0 ± 2.6	47.8 ± 5.1	48.5 ± 3.1	46.5 ± 5.5	47.5 ± 3.1	45.8 ± 4.0	46.9 ± 4.6
	% GLU II	55.0 ± 2.6	52.2 ± 5.1	51.5 ± 3.1	53.5 ± 5.5	52.5 ± 3.1	55.2 ± 3.0	53.1 ± 4.6
	% Lys I & II	-	-	-	-	-	-	-
iPLG-OMP	PL:Act (U/mL)	<0.005	<0.005	<0.005	<0.005	<0.005	<0.005	<0.005
	%PLG-band	96.7 ± 1.6	95.0 ± 1.8	95.3 ± 2.1	93.9 ± 2.4	95.8 ± 2.6	88.6 ± 2.6	94.8 ± 2.2
	% GLU I	43.5 ± 1.6	45.3 ± 0.4	45.4 ± 0.4	45.4 ± 4.6	45.5 ± 0.6	44.7 ± 1.3	43.3 ± 1.0
	% GLU II	56.6 ± 1.6	54.7 ± 0.4	54.3 ± 0.8	54.6 ± 4.6	54.5 ± 0.6	55.3 ± 1.3	56.5 ± 1.3
	% Lys I & II	-	-	-	-	-	-	-

**Table 3:** Dimensional analysis of aerosols obtained by the nebulization of PLG-OMP, iPLG-OMP and saline solutions (NaCl 0.9% was used as a reference). Median diameter value (dv_(50)_), distribution width (SPAN = (dv_(90)_ − dv_(10)_)/dv_(50)_), percentage of particles that could reach the lower airways (%V < 5 µm) and percentage of particles compatible with tracheobronchial distribution (%V < 10 µm). Student’s *t*-test was applied to compare the data within each nebulization setting.

	J2			J3			US		
	PLG-OMP	iPLG-OMP	NaCl	PLG-OMP	iPLG-OMP	NaCl	PLG-OMP	iPLG-OMP	NaCl
dv_(50)_ (μm ± sd)	9.2 ± 1.0	8.1 ± 0.2	8.79	5.6 ± 0.5	5.5 ± 0.1	5.5	4.9 ± 0.3	5.1 ± 0.1	5.5
SPAN (value ± sd)	2.2 ± 0.2	2.1 ± 0.1	2.17	1.5 ± 0.2	1.6 ± 0.1	1.7	78.8 ± 5.0	76.8 ± 6.1	75.7
%V < 5 µm (% ± sd)	24.6 ± 5.1	27.0 ± 1.3	25.39	41.9 ± 6.5	43.9 ± 1.6	43.51	50.6 ± 2.9	48.7 ± 1.1	43.91
%V < 10 µm (% ± sd)	53.8 ± 5.0	60.5 ± 1.7	56.53	85.5 ± 2.7	85.1 ± 2.1	83.92	78.4 ± 0.6	77.1 ± 1.1	75.92
